# Adulteration of Food and Food Products1Read at the eighty-third semiannual meeting of the Medical Society of the County of Erie, June 28, 1904.

**Published:** 1904-09

**Authors:** John H. Grant

**Affiliations:** 715 Mutual Life Building, Buffalo, N. Y., Assistant Commissioner of Agriculture, State of New York


### Adulteration of Food and Food Products.[1]

By JOHN H. GRANT, M. D., Buffalo, N. Y.,
Assistant Commissioner of Agriculture, State of New York.

THE title of this paper is in itself a large subject and one that it would be impossible to dwell upon except in a general way during the limited time at my disposal. The law governing the manufacture, keeping for sale and selling food and its products is in its infancy in New York State. About a year ago an amendment to the agricultural law was passed placing the enforcement against food adulterations, false labeling, branding and the like, in the hands of the department of agriculture, the term food including all articles of food, confectionery and condiments, whether simple or compound, used for human consumption.

Confectionery is to be deemed adulterated if it contains terra alba, barytes, talc, chrome yellow or other mineral substances or poisonous flavors or colors, or other ingredients deleterious or detrimental to health. The law also declares food to be adulterated: first, if any substance or substances have been mixed or packed with it so as to lower or injuriously affect its quality or strength; second, if any substance or substances has or have been substituted wholly or in part for the article; third, if any valuable constituent of the article has been wholly or in part abstracted; fourth, if it contain any added poisonous ingredient or any ingredient which may render such article injurious to the health of the person consuming it; fifth, if it consists in whole or in part of a filthy, decomposed, or putrid animal or vegetable substance, or if it is the product of a diseased animal or one that has died otherwise than by slaughter; and provided, that an article of food

1. Read at the eighty-third semiannual meeting of the Medical Society of the County of Erie, June 28, 1904.

which does not contain any added poisonous or deleterious ingredients, shall not be deemed to be adulterated or misbranded in the following instances—namely, articles labeled, branded or tagged so as to plainly indicate that they are mixtures, compounds, combinations, imitations or blends and that the labels, brands or tags show the character and constituents thereof.

It will be seen that the principal object of this law is to prevent deception; it does not prevent sophisticated articles from being sold as compounds so long as the label shows the character and constituents thereof—and provided always that it contains no added poisonous ingredient. This latter proviso is important as will be seen further on. All the states and territories of the Union, even Alaska, have some laws looking towards the prohibition, detection of and penalties against the manufacture and sale of imitation and adulterated foods. Of course, the officers charged with the enforcement of the New York State law would prefer more specific provisions,—for example, a fixed standard for each article, making the detection of food frauds merely a matter of simple chemistry, that is, of analysis and comparison. It is very easy to show that an adulterated cider vinegar, for instance, contains less than 4½ per cent. of acetic acid; but, it is not so easy to prove in court that a substance has been mixed with the vinegar so as to reduce or lower, or injuriously affect its quality or strength, or that it contains ingredients injurious to the health of the person consuming it. This lack of a fixed standard, in my judgment, is the weak point in our law, as both sides would bring into court expert testimony, confusing the jury and resulting in many instances in no cause of action or acquittal of the defendant.

There is now a cider vinegar case before the court of appeals in which the manufacturer admitted adding to every eight barrels of vinegar, one barrel of hydrant water, on the plea that the original stock was too strong in acidity and the added water lowered it in strength to comply with the state law. This argument swayed the lower court and the case was dismissed. But how falacious this argument! The addition of boiled cider or cider itself would have inhibited the production of acid beyond the desired strength, but this would not answer the purposes of the manufacturer. The profit would not be so great.

In October last we began here in Buffalo to enforce the provisions of the pure food law on manufacturers and wholesalers, and later on a few retailers. We commenced first on honey and maple syrup; about fifteen different brands or samples of so-called pure honey were analysed, of which ten were found adulterated, being made up principally of from 12½ to 90 per cent.

of glucose. In some samples no honey was found except that contained in a small piece of honey comb immersed in the syrup; in one case the honey comb was found to be artificial.

Of maple syrup, a number of samples were taken of different brands; five of them were adulterated, some containing no maple syrup at all, the flavor being given by some extract of bark, and all were made up largely of cane sugar. This is one of the most difficult substitutions to detect, as the polarisation of both sugars is the same; but, thanks to the investigations of Dr. Herbert M. Hill and his assistant, Dr. Willet H. Mosher, of this city, as well as chemists connected with the department of agriculture in Pennsylvania, certain reactions take place in true maple sap not found in mixtures or compounds simulating the same.

We next turned our attention to unfermented grape juices and out of some four different brands, two of them claiming on the label to have no antiseptic therein, were found to contain added preservatives,—salicylic acid and benzoic acid,—enough in a bottle, especially of the former, to irritate a sensitive stomach,—a condition for which these juices are often prescribed, and the presence of the preservative defeating the object of the prescriber. The manufacturers of these preserved grape juices were only too anxious to settle the cases without publicity, and promptly paid the penalties incurred,—$50.00 for each bottle.

Our next samples were of so-called jams, a number of which were found to be sophistications; such as grass seeds worked up in glucose for raspberry jam, colored with coal-tar dye, and preserved with comparatively large quantities of salicylic acid. Our attention was then directed to fresh sausage meats and ground beef or hamburger steak. A considerable number of samples was taken, and all but one were found to contain sulphites, lime or sodium, put in, we were told, to give the meat a rich red color and to keep it for a week or more without ice.

During our tours of inspection we ran across a filthy looking cellar in Scott street, Buffalo, in which a party was found manufacturing tomato catsup. The windows of the cellar were covered with soot and cobwebs; in the center was a large iron caldron, from its appearance apparently never cleaned, in which was stewing a mess. Upon investigation it was found that the material stewing in the caldron was made up of rotten apples bought at the various markets for a trifling sum. The seeds and cores were removed by straining, and a piece of common glue, of which there was a hogsheadful in the cellar, was then dissolved in the mess, to give it consistence, red aniline coal-tar dye being added to give a beautiful scarlet color. The stuff was then bottled and labeled with fancy colored labels on which was depicted a large ripe tomato, and the material was ready for sale as pure tomato catsup. This place has been abandoned, the party not waiting for prosecution, but silently stealing away to some other locality probably, where he may perhaps avoid the inquisitive eyes of the inspectors.

Another matter, interesting to druggists, is the keeping for sale or selling so-called butter color, put up by a prominent firm in Vermont. Several samples were taken from druggists in Buffalo which, upon being analysed, were found to be made up of cottonseed oil in which a coal-tar color of the azo group was dissolved. This is a dangerous preparation, although ordinarily the small quantity used in the butter does probably little immediate harm; but ignorant persons, handling it in dairies or creameries, who have no knowledge of drugs, may be and are often careless, several instances being on record where persons have died from its effects in producing acute gastritis. One case is recorded recently of eating the butter by a woman who was made deathly sick; and a case occurred here in Buffalo, where a boy in the night got hold of a bottle, drank considerably less than an ounce, and the next morning was found dead. There are many harmless vegetable colors, such as anatto, turmeric, litmus and cochineal on the market that are safe, and there is no excuse except profit for this Vermont preparation to be used as it is.

The question of preservatives and coloring agents in foods is now one of the most important we have to deal with. Antiseptics for our purposes may be divided into two classes: first, those admittedly harmless, such as common salt, saltpetre, sugar, spices, and the like. These have been used since time immemorial and their use has been recognised as legitimate. The second class embraces the so-called chemical preservatives, of which may be mentioned salicylic and benzoic acids and their salts, boric acid and borax, formaldehyde, saccharin, sucrol, sulphurous acid and its salts, abrastol, betanaphthol, some of the fluorine compounds and others. The application of these preservatives to food products is of comparative recent date. We all remember the embalmed beef scandal in our army during the Spanish-American war. In the opinion of the writer their use is a menace to public health and longevity. These chemiical preservatives, it might be well to state, are used for one of the following purposes: first, to prevent fermentative or putrefactive processes in the article of food after it is placed on the market; second, for the purpose of arresting decay in raw materials before manufacture; and, third, for the purpose of disinfecting tainted raw substances in order that the unsanitary material may be sold in the place of sound, wholesome food-stuffs.

To explain the present almost universal use of preservative and coloring agents in foods and food products it is only necessary to say that competition among manufacturers and retail dealers with each other is greatly responsible. Although the application of the principles of chemistry to the various industries, and to soils and fertilizers, have aided wonderfully in the development of food-raising industries, the same art has also developed means of sophistication to replace and compete with these industries; especially has the manufacturer availed himself liberally of the synthetic productions of chemistry as in the use of preservatives, coloring matter and flavors. It is to be remembered that the processes of digestion in the human system are essentially fermentative, brought about by means of a series of enzymes, and any agent that will prevent fermentation in a food product will also render it indigestible, inhibiting the digestive process in the stomach and intestinal tract, until removed or absorbed by the system. Nearly everyone of our modern food products may be found in the market carrying one or more chemical preservatives.

All this becomes a matter of grave concern. Think, for a moment, what a mixture or combination of chemicals a person is apt to put into his or her stomach during an ordinary meal! Soup and meat contain, perhaps, sulphurous acid; milk and cream may have formaldehyde. A physician in this city tells me that he always tests his cream by giving some to the family cat; if the cat sniffs at it and walks away he feels sure it contains formaldehyde. Apple butter, or honey, is made in great part of colored glucose; pudding is flavored with lemon extract made of oil of lemon grass with yellow aniline or a vanilla extract made with Tonka bean or coumarin; butter is colored with an azo dye; coffee to cover up damage or inferiority has been glazed with dextrine and starch and beer or wine contains salicylic or benzoic acid. Well might it be said, what shall it avail a man to gain a full dinner pail if he cannot digest the contents?

What has been said of preservatives also holds good of coloring agents. Many of the latter are also preservatives. Generally their use in foods is to simulate the color of the particular article sought to be imitated, or to cover up by means of the bright colors imparted, out and out adulterations, substitutions or damaged or inferior goods.

As an evidence of the realisation of this danger of chemical preservatives and coloring matter a jury in the supreme court in

Buffalo only a few weeks ago rendered a verdict of $75.00 fine against a milk dealer who had put formaldehyde in his cream, and $50.00 against a grocer who was selling glucose for that wonderful transformation, the nectar of flowers,—the product of the honey bee. These facts are encouraging indeed.

Another matter of special interest to physicians is the recent enactment of our legislature forbidding the unauthorised use of a certified label of milk. Its enactment was demanded as it was found that unscrupulous dealers were taking milk of any kind, marking it "certified" and by so doing imposing on the consumer, as well as the producer. It will hereafter be unlawful to certify to milk except under the authority of a competent person. Some may not understand what is meant by the term certified milk; it is milk produced on the principle that the natural fluid just as it comes from the healthy cow is in the best possible condition for human food if only we can keep it uncontaminated by the entrance of extraneous impurities, and having as a bacteriological standard not more than 30,000 bacteria to the cubic centimeter. This standard is reached by perfect sanitary conditions, cleanliness of stables, cows and milkers.

In closing this subject, I would not wish you to think me pessimistic, for I am optimistic as to the future benefits to be derived in the continued enforcement of the pure food law. Like all new laws it is not perfect; its practical workings will discover its weak spots; the courts will construe, but these constructions will, no doubt, reasoning from past experiences in the execution of our dairy laws, meet the requirements of the people and at the same time will not be unreasonable or harsh on the producer or dealer.

715 Mutual Life Building.

